# The Effect of Sanitizers on Microbial Levels of Chicken Meat Collected from Commercial Processing Plants

**DOI:** 10.3390/ijerph16234807

**Published:** 2019-11-29

**Authors:** Kapil Chousalkar, Sarah Sims, Andrea McWhorter, Samiullah Khan, Margaret Sexton

**Affiliations:** 1 School of Animal and Veterinary Science, The University of Adelaide, Adelaide 5371, Australia; Sarah.Sims@sahmri.com (S.S.); andrea.mcwhorter@adelaide.edu.au (A.M.); samiullah.khan@adelaide.edu.au (S.K.); 2 Primary Industries and Regions, Adelaide 5065, Australia; margaret.sexton@sa.gov.au

**Keywords:** chicken meat, sanitizers, *Campylobacter*, *Salmonella*

## Abstract

Chicken meat can potentially become contaminated with bacteria at the processing plant. In Australia, there is currently a lack of knowledge on the parameters and indications of use of non-chlorine based treatments in the chicken meat processing plants. Chlorine is widely used as a sanitizer in Australian chicken meat processing plants but due to occupational health and safety concerns and consumer perception, there is a need to identify alternative sanitizers. This study aimed to assess the efficacy of four different sanitizers in reducing the microbial load from naturally contaminated chicken meat carcasses collected from the processing plants in South Australia. There was a significant variation in a load of *Campylobacter* and total viable count (TVC) between samples collected from two different processing plants and within carcass batches collected from the same plant that was tested during the study. All sanitizers generally reduced the load of *Campylobacter* on chicken meat carcasses. Treatment with acidified sodium chlorite significantly reduced the level of *Salmonella enterica* serovars at all temperatures tested during this study. These findings are helpful to the industry for selection of the appropriate sanitizers. Findings are also useful for the regulatory authorities in Australia for providing approval for the use of sanitizers.

## 1. Introduction

Worldwide, the per capita consumption of chicken meat is increasing [1]. Over the past 20 years, in Australia, the consumption of chicken has increased from 30.9 kg per person in 2000 to 43.9 kg/person in 2018 [2]. Current models project further increases and it has been calculated that the average chicken meat consumption will be 51.5 kg/person in 2022 [3].

Sixty-nine percent of Australian chicken meat products are sold raw with no further processing [3]. Raw or undercooked chicken meat or meat products, however, are potential sources of pathogenic bacterial species, *Campylobacter* and *Salmonella*. *Campylobacter* and *Salmonella enterica* serovars are the leading causes of foodborne human gastroenteritis in Australia [4]. In the US and Australia, the consumption of contaminated poultry meat has been attributed for the majority of food-related illness [5,6,7,8]. In Australia, the presence of *Campylobacter* and *Salmonella* on raw post-slaughter meat is estimated at 84.3% and 22.1%, respectively [9]. There is a linear correlation between bacterial prevalence on chicken meat at the final stage of processing and the cases of illness [10]. A 2010 survey found similar bacterial prevalence post-production on whole carcasses from South Australia, New South Wales, Queensland, and Victoria [9]. A separate survey of retail chicken portions and carcasses reflected similar values [11]. Despite the implementation of the Poultry Processing Standards for Poultry meat in 2012, there has been no significant reduction in the number of food poisoning cases [4,12,13,14]. 

Australian chicken meat farms and processing plants use a number of intervention strategies to reduce or control the load of foodborne pathogens throughout the food supply chain. Chicken meat can potentially become contaminated with bacteria during transportation, slaughter, evisceration, partitioning, and packing [15,16]. Carcasses are commonly sanitized in processing plants through a series of washes using chlorinated water to reduce surface contamination. The Australian chicken meat industry has widely used chlorine for poultry processing but due to occupational health and safety concerns and consumer perception, there is a need to identify alternative sanitizers. The European Union currently does not permit the use of sanitizers for chicken meat [17]. Globally, there are a number of sanitizers that have been trialed and used in washing and chilling of chicken carcasses to reduce foodborne pathogens; however, some of these sanitizers have either not been registered/approved or trialed in processing plants in Australia and in some other countries. Apart from the recommendations in the Australia New Zealand Food Standards Code, there are no other definitive standards in Australia for the use of a wide range of other sanitizers on chicken meat in poultry plants. Section 1.3.3, Schedule 18 [10], of the Australia New Zealand Food Standards Code allows sanitizers such as chlorine, peroxyacetic acid (PAA), and sodium hypochlorite for use as a processing aid for washing of all foods; hence, as a starting point, along with chlorine, acidified sodium chlorite (ASC), PAA, and PoultrypHresh (CMS Technology, Bridgewater, NJ, USA) were specifically selected in this study.

This study aimed to assess the efficacy of four different sanitizers at different temperatures at reducing the load of *Campylobacter* and *Salmonella enterica* serovars from the contaminated chicken meat carcasses collected from the processing plants. The four sanitizers were chlorine, acidified sodium chlorite (ASC), PoultrypHresh, and peroxyacetic acid (PAA). The efficacy of sanitizers were tested at various temperatures that were relevant to processing plants in Australia. The concentration of chlorine, ASC, and PAA was selected based on previous studies [10,18,19]. The concentration of PoultrypHresh was used as described by the manufacturer. To mimic the field/processing plant conditions, the exposure time of the sanitizers was selected based on the current practices in the Australian processing plants.

## 2. Materials and Methods

### 2.1. Chicken Meat Samples

Chicken carcasses were obtained from two commercial poultry processing plants. To determine an optimal sample size, 15 chicken meat carcasses from pre- and post-wash processing steps, respectively, were obtained and tested for *Campylobacter* spp. as described below. Using this preliminary data, power calculations were performed with the binomial primary end point (*Campylobacter* positive/negative results in this case). Each sanitizer was tested using 30 chicken meat carcasses and each experiment was repeated twice. Altogether, 240 chicken meat carcasses (big birds) were tested in this study. All chickens were from intensive production systems (deep litter) and the average carcass weight was 2.2 kg. For each experiment, chicken meat carcasses (n = 30) were obtained immediately prior to the inside outside wash step. Samples were placed on ice and transported to the laboratory for experiments. Each sanitizer was tested independently and each experiment was repeated twice. Each carcass was immersed into a tank containing 13 L of sanitizer solution. 

### 2.2. Sample Processing, Isolation, and Enumeration of Bacteria

In each experiment, 30 chicken carcasses prior to inside outside wash step were collected from a processing plant in large sterile bags. Chickens were weighed and the deep muscle breast temperature was recorded within 20 min of arrival in the laboratory. Individual carcasses were subsequently placed in a whirl pack bag (ThermoFisher Scientific, Scoresby, Australia) and washed with massaging for 2 min in 200 mL buffered peptone water (BPW; ThermoScientific, Oxoid, Scoresby, Australia). Two mL of the BPW wash were spread plated onto five modified charcoal-cefoperazone-deoxycholate agar (mCCDA) (ThermoScientific, Scoresby, Australia) plates (400 µL per plate) and incubated at 42 °C with 10% CO_2_ for 48 hours to assess direct *Campylobacter* spp. counts. From the initial 200 mL BPW wash, 40 mL was incubated overnight at 37 °C. Then, 100 µL of the incubated BPW was transferred into 10 mL Rappaport Vassiliadis soya peptone broth (RVS, ThermoScientific, Oxoid, Scoresby, Australia) and incubated overnight at 42 °C for selective growth of *Salmonella enterica* serovars. A loop-full of the RVS broth was streaked on to xylose lysine deoxycholate agar (XLD; ThermoScientific, Oxoid, Australia) plates. Suspected *Salmonella* colonies were subcultured onto Brilliance *Salmonella* agar (ThermoScientific Oxoid, Scoresby Australia) for confirmation. To quantify the total viable counts (TVC) in the carcass wash, the carcass wash was diluted 10-fold and plated on nutrient agar. The limit of detection for TVC was 0.25 colony forming units (CFU)/cm^2^ of chicken carcass. The plates were incubated overnight at 37 °C and colonies were recorded in colony forming units (CFU). For *Campylobacter* spp. the limit of detection was 10 CFU/mL of rinsate. 

Following the initial BPW wash, 5 carcasses each were placed into six different treatment groups, water and sanitizer wash each at 5 °C, 15 °C, and 22 °C (Table 1). Carcasses were placed into large containers filled with diluted sanitizer and agitated continuously for the entire treatment period (Table 1). Carcasses were then removed and placed in a sterile bag and rinsed as with BPW. The miniaturized Most Probable Number (MPN) method described by [20], was used to determine the *Salmonella* load in culture-positive samples. *Campylobacter* and *Salmonella* counts as well as the TVC were interpreted per square centimeter of carcass as per the Australian standard [21]. Briefly, surface area of a whole chicken carcass in square centimeters was calculated by the following formula, 0.87 m + 635 (m = total mass in grams) and the microorganisms per square centimeter of surface area from the rinse fluid was calculated using the following formula,

Colony forming units (CFU)/cm^2^ = Number of colonies × volume of rinse fluid (200 mL).

#### Surface Area of Poultry Meat

Efficacy experiments were conducted twice for each sanitizer. The concentration of each chemical, carcass weight, and pH conditions are outlined in Table 1.

The data obtained (microbial reductions and microbial counts) were compared for significant differences (*p* < 0.05) using two-way analysis of variance (ANOVA) and the Fishers protected least significant difference (PLSD) test using GraphPad Prism version 8 (San Diego, CA, USA). The data were normally distributed. 

## 3. Results

For sample size calculation, 15 pre- and post-washed samples were obtained from a processing plant to determine total *Campylobacter* load at each step. Prior to spin chilling 15/15 samples (100%) were *Campylobacter* spp. positive while 14/15 (93%) post-chill carcasses were positive. Based on these results, in order to detect a 7% difference between pre- and post-wash treatment, five carcasses were required for each treatment group (α = 0.05, β = 0.1, and power = 0.95). Each sanitizer was tested twice (Six treatments for each sanitizer × five samples × 2 replications). Upon receipt from the processing plant all carcasses were pre-washed with BPW to characterize the initial bacterial load and then treated with sanitizers for the assessment of their efficacy.

### 3.1. Effects of Chlorine on the Bacterial Count

**Figure 1:**
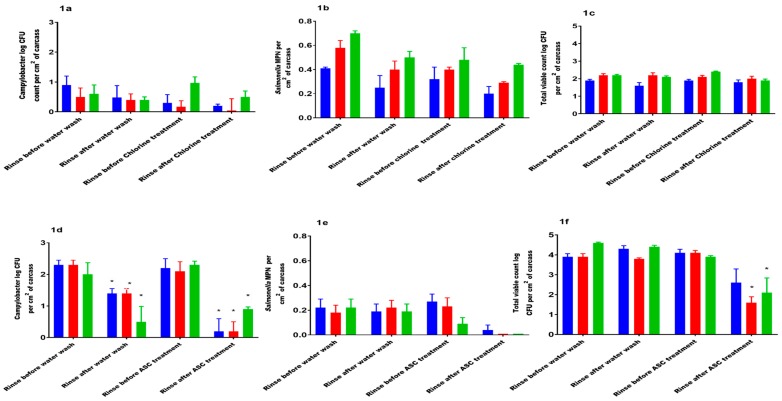
(**a**) *Campylobacter* load on chicken meat carcass pre- and post-chlorine treatment (4–8 ppm), 20 min agitation; (**b**) *Salmonella* count on chicken meat carcasses pre- and post-chlorine treatment (4–8 ppm), 20 min agitation; (**c**) total viable count (TVC) load on chicken meat carcasses pre- and post- chlorine treatment (4–8 ppm), 20 min agitation; (**d**) *Campylobacter* load on chicken meat carcasses pre- and post-acidified sodium chlorite (ASC) treatment (900 ppm), 20 s agitation; (**e**) *Salmonella* MPN pre- and post-acidified sodium chlorite treatment (900 ppm), 20 s agitation; (**f**) TVC pre- and post-acidified sodium chlorite treatment (900 ppm), 20 s agitation. For all figures, blue color column represents 4 °C, red color column represents 15 °C, green color column represents 22 °C. * = significant difference between treatment groups; CFU = Colony Forming Units; MPN = Most Probable Number.

Following chlorine treatment, the *Campylobacter* count and TVC were reduced at all temperatures (4 °C, 15 °C, and 22 °C); however, this was not significant. Washing with water also resulted in reduction of *Campylobacter* count at 4 °C and 15 °C but this reduction was not significant. The water wash at 22 °C did not result in a reduction of *Campylobacter* or TVC levels. No reduction in the level of *Salmonella enterica* serovars after either the water or the chlorine wash was observed. Overall, no significant effect of temperature was observed (Figure 1a–c). In this study the chlorine treatment at the tested exposure times resulted in reduction of TVC by log 0.1 at 4 °C and 15 °C, and log 0.5 at 22 °C.

### 3.2. Effects of Acidified Sodium Chlorite (ASC) on Bacterial Count

Washing chicken meat carcasses with plain water resulted in a significant reduction of *Campylobacter* counts (*p* = 0.006) at all temperatures. No significant reduction in the prevalence of *Salmonella enterica* serovars after washing with water was observed. Significant reductions in the *Campylobacter* count were observed when the carcasses were treated with ASC at all temperatures. There was a reduction in TVC in ASC-treated carcasses at all temperatures, but this reduction was significant only at 15 °C (*p* = 0.002). No significant reduction of *Salmonella* prevalence was observed post ASC treatment. Overall, there was a significant treatment effect of ASC on the *Campylobacter* count (*p* < 0.001), *Salmonella* MPN (*p* < 0.001), and TVC (*p* < 0.001, Figure 1d–f). In this study the ASC treatment at the tested exposure times resulted in reduction of TVC by log 1.5 at 4 °C, log 2.5 at 15 °C, and log 1.8 at 22 °C.

### 3.3. Effects of PoultrypHresh on Bacterial Count

During this trial, the washing of carcasses with plain water resulted in a reduction of *Campylobacter* at all temperatures but the reduction was significant only at 15 °C (*p* = 0.04). After the water wash, the TVC was significantly reduced at 4 °C (*p* = 0.01) and 15 °C (*p =* 0.01). There was no significant difference in *Salmonella* prevalence prior to or after water wash. 

**Figure 2:**
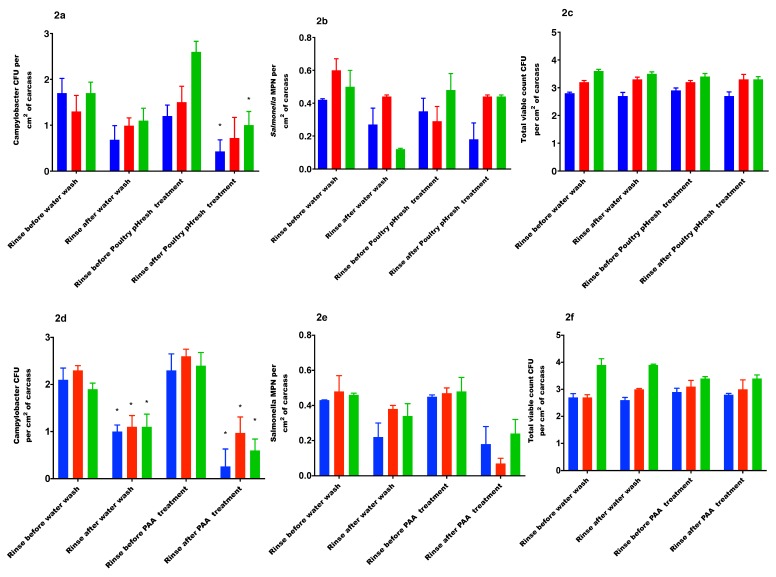
(**a**) *Campylobacter* load on chicken meat carcasses pre- and post-PoultrypHresh treatment (pH 1.4–1.6), 12 s agitation; (**b**) *Salmonella* MPN pre- and post-PoultrypHresh treatment (pH 1.4–1.6), 12 s agitation; (**c**) TVC on chicken meat carcasses pre- and post-PoultrypHresh treatment (pH 1.4–1.6), 12 s agitation; (**d**) *Campylobacter* load on chicken meat carcasses pre- and post-PAA treatment (200 ppm), 20 min agitation; (**e**) *Salmonella* MPN pre- and post-PAA treatment (200 ppm), 20 min agitation; (**f**) TVC on chicken meat carcasses pre- and post-PAA treatment (200 ppm), 20 min agitation. For all figures, blue color column represents 4 °C, red color column represents 15 °C, green color column represents 22 °C. * = significant difference between treatment groups.

PoultrypHresh treatment resulted in significant reductions of *Campylobacter* counts at all temperatures. There was a reduction in TVC after PoultrypHresh at all temperatures, but this was not significant. No significant difference in *Salmonella* prevalence was observed pre- or post-PoultrypHresh treatment. Overall, a significant effect of PoultrypHresh treatment on the *Campylobacter* count (*p* < 0.001) was observed. (Figure 2a–c). In this study the PoultrypHresh treatment at the tested exposure times resulted in a reduction of TVC by log 0.2 at 4 °C, and log 0.1 at 15 °C and 22 °C.

### 3.4. Effects of Peroxyacetic Acid (PAA) on the Bacterial Count

The level of *Campylobacter* was significantly reduced when the carcass samples were rinsed with plain water at all temperatures (*p* = 0.007). 

Treatment of carcasses with PAA resulted in a reduction of TVC at all temperatures but this treatment effect was not significant. Neither PAA nor water significantly reduced the *Salmonella* prevalence (number of positive samples before and after the treatment). Treatment of carcasses with PAA resulted in significant reductions of *Campylobacter* at all temperatures (Figure 2d–f). In this study the PAA treatment at the tested exposure times resulted in a reduction of TVC by log 0.1 at 4 °C and 15 °C and no reduction at 22 °C.

## 4. Discussion

Poultry meat is considered to be a significant source for human campylobacteriosis in Australia and a national baseline survey has revealed that there is a high prevalence of *Campylobacter* on poultry meat carcasses at both the end of processing and at the point of retail [9]. It is important to look at the critical control points in the processing plant. It has been suggested that a 2-log reduction in the level of *Campylobacter* spp. can significantly lower the risk of human campylobacteriosis. Internationally, various bactericidal chemicals have been tested as processing aids with the aim of reducing pathogenic bacteria on processed poultry carcasses [19,22,23]. In this study, four sanitizers were tested at different temperatures (22 °C = ambient temperature, 15 °C = prechilling, 4 °C = chilling) relevant to Australian poultry meat processing conditions.

Chlorine is widely used for the reduction of foodborne pathogens in processing plants in Australia. In the present study, chlorine resulted in a reduction in the total count of *Campylobacter*. Our results are in agreement with Berrang et al. [24]; however, another study [25] reported no significant reduction in *Campylobacter* levels after post-chill chlorine treatment. It is well established that the efficacy of chlorine is dependent on the level of organic material, pH, load of bacteria on pre-chill carcasses, and the amount of free chlorine available [26]. In this study, temperature was not associated with a significant effect of chlorine in the reduction of *Campylobacter* on treated carcasses. Although the concentration of chlorine used in this study was lower than previous studies [27], the *Campylobacter* reduction was achieved at all the tested temperatures (reductions by log 0.1 at 4 °C, log 0.06 at 15 °C, and log 0.4 at 22 °C). It is important to note that these reductions were not significant at any of the tested temperatures. Further work is required to look at the effect of this concentration at processing plants.

Our findings regarding the effect of ASC on the reduction of *Campylobacter* is in agreement with previous findings [19,27]. Previous studies have also reported minimal effects of ASC in reducing the level of *Salmonella* and *Campylobacter* spp. [22]. In the present study, ASC was acidified using citric acid. It has previously been reported that citric acid in different concentrations can have effects on the color of the chicken fillets [28]. Although the sensory properties of chicken carcasses were not investigated in depth during this study, change of color of the carcass was temporary. The *Campylobacter* reduction was achieved at all the tested temperatures (reductions by log 2 at 4 °C and 15 °C, and log 1.4 at 22 °C). It is, however, necessary to test the different concentrations of citric acid and ASC on the level of reduction of *Salmonella* and *Campylobacter*. 

Our findings on the effect of PoultrypHresh on the level of *Campylobacter* is in agreement with previous investigation [29]. No effects on the appearance of the meat was observed in treated carcasses. The previous study [29] reported a 3-log reduction after 25 s of exposure. In the present study, *Campylobacter* reduction was achieved by PoultrypHresh treatment at all the tested temperatures (reductions by log 0.8 at 4 °C, log 0.8 at 15 °C, and log 1.6 at 22 °C). Further experiments are required to establish an exposure response time on the level of bacterial reduction.

Several studies have reported on the efficacy of PAA in reducing the level of *Campylobacter* and *Salmonella* [30,31]. In our study, although there was a reduction in the level of *Campylobacter* load, in general the reduction was significant only at 15 °C (log 1.7 reduction) compared to 4 °C (2.1 log reduction) and 22 °C (1.8 log reduction). This could suggest that the efficacy of PAA is temperature dependent. In this study, only one concentration of PAA was tested and further studies are required to test various concentrations at different temperatures.

The total viable count on chicken meat carcasses has been used as an indicator of shelf life of the product [32,33]. Our results regarding the temperature-dependent effects of sanitizers on the level of TVC is in agreement with previous studies [33,34]. This study also confirms that the sanitizer application temperature is an important factor to consider when pathogen reduction protocols are developed for poultry carcasses. In this study, the ASC treatment at 15 °C resulted in 2-log reduction in *Campylobacter* and TVC and resulted in significant reductions in *Salmonella* level (reduction by log 0.1). ASC treatment was also effective at 4 and 22 °C. ASC can be a possible alternative to chlorine treatment in the processing plant, however, in-field processing plant trials are essential. In addition, further studies are necessary to test different concentrations and exposure times of ASC for reduction of *Campylobacter*, *Salmonella,* and TVC. 

Australian Standards for processing of meat in Australia are still prescriptive. This project is designed to provide a demonstration of the actions and evidence required to support application to the controlling authorities (and possibly customers) that the change to the process, or in this case specifically the sanitizers used, is at least equivalent to those prescribed in the Standard and results in a consistent, safe, and suitable product. The data produced from this work is useful for both industry and regulatory authorities for selection and or approval of alternative chemicals, other than chlorine. Chlorine has been widely used in Australian processing plants due to its efficacy and the cost. In this study, although there was a reduction in *Campylobacter* and TVC, the reduction levels were not significant at any of the tested temperatures. On the other hand, ASC, PAA, and PoultrypHresh were able to significantly reduce the level of *Campylobacter* and TVC at certain temperatures. There is an option for the industry to adopt the use of sanitizers other than chlorine, however further work on the effects of these sanitizers in field conditions is necessary. It is important to note that none of the sanitizers were able to completely eliminate the prevalence of *Campylobacter* and *Salmonella*. Chlorine has occupational health concerns. Given that there is lack of information on established limits of concentration for PAA and ASC in Australian conditions, further studies are essential to establish the limit of concentration, exposure time, and subsequent organoleptic effects. 

Our results regarding the variation in the level of *Campylobacter* in between and within the processing plant is in agreement with previous reports [35,36], which suggested that the *Campylobacter* contamination of carcasses does not occur homogenously. It has been suggested that bacterial cells injured by sanitizers can be recovered during chilling [37]. It is possible that such “injured cells” either enter into viable but noncultural state or form biofilms and resuscitate in a favorable environment. Further studies are necessary to understand the phenotypic and genomic changes in *Campylobacter* after exposure to different sanitizers. Detailed investigation on the effects of these sanitizers on the sensory properties of the chicken meat carcass is also necessary. The findings of this study are helpful to the industry for selection of the appropriate sanitizers for its use on a wider scale. The information from this work is also useful for the regulatory authorities in Australia for providing approval for the use of sanitizers.

Globally, protecting public health by controlling foodborne pathogens in chicken meat continues to be a challenge to both industry and regulators [38]. In Australia, the majority of the processing plants use chlorine as a single intervention to minimize the risk of foodborne pathogens. The results from this study indicated that ASC could be used as an effective intervention due to its efficacy in reducing *Campylobacter*, *Salmonella,* and the TVC. But its cost and the necessity to combine it with generally recognized as safe acids need to be considered [38]. PAA was effective in reducing *Campylobacter* and *Salmonella* but not the TVC. Previously, the European Food Safety Authority found a reduction in the prevalence of foodborne pathogens after PAA addition during the chilling step, although the effects of PAA on TVC were not reported [39]. However, a better understanding of the impact of ASC and PAA at different stages and temperatures in the processing plant is needed. Moreover, further studies on a combination of interventions in Australian settings will also be useful. 

## 5. Conclusions

There was a significant variation in the load of *Campylobacter* and TVC between processing plants and within carcass batches that were tested during the study. Based on the *Campylobacter* isolation results from pre- and post-treatment groups, five carcasses are sufficient for testing the effects of sanitizers on the level of *Campylobacter*. All sanitizers reduced the load of bacteria (TVC, *Campylobacter,* and *Salmonella* counts) on chicken meat carcasses. In this study, ASC treatment was the most effective and can be used widely. ASC treatment for 20 s resulted in log 2 reduction of *Campylobacter* and TVC at 15 °C. Hence, this treatment can be used for sanitizing carcasses in the processing plant. ASC can also be used effectively at 22 °C and 4 °C. The efficacy of sanitizers was temperature dependent, hence, control of temperature is critical. Further studies are required to test the efficacy of different concentrations of sanitizers at different temperatures. Apart from chlorine, there are other sanitizers such as ASC that could be used for sanitizing carcasses in the Australian processing plants but in-plant studies are required.

## Figures and Tables

**Table 1:** List of sanitizers used in this study along with carcass weight, temperature, and exposure times at various temperatures.

Sanitiser	Concentration	pH Range	Average Carcass Weight (kg)	Average Carcass Temperature	Agitation Time		
					4 °C	15 °C	22 °C
Chlorine	50 ppm total available chlorine	5.5–6.5	2.20	26.1 °C	20 min	20 min	20 min
PoultrypHresh	Added to adjust the desired pH	1.4–1.6	2.26	27.1 °C	12 s	12 s	12 s
peroxyacetic acid (PAA)	200 ppm	2.5–2.7	2.26	27.1 °C	20 min	20 min	6 s
Acidified Sodium Chlorite	900 ppm	2.5–2.6	2.28	26.7 °C	20 s	20 s	20 s
